# Trends and risk factors for neonatal mortality in Butajira District, South Central Ethiopia, (1987-2008): a prospective cohort study

**DOI:** 10.1186/1471-2393-14-64

**Published:** 2014-02-11

**Authors:** Muluken Gizaw, Mitike Molla, Wubegzier Mekonnen

**Affiliations:** 1 Department of Preventive Medicine, School of Public Health, College of Health Sciences Addis Ababa University, Addis Ababa, Ethiopia; 2 Department of Reproductive Health and Management, School of Public Health, College of Health Sciences Addis Ababa University, Addis Ababa, Ethiopia

**Keywords:** Neonatal mortality, HDSS, Butajira district

## Abstract

**Background:**

Child mortality is an important indicator of a country’s developmental status. Neonatal mortality and stillbirth shared a higher proportion of child deaths. However, in developing countries where there is no civil registration and most deliveries occur at home, it is difficult to measure the magnitude of neonatal mortality. Data from continuous demographic surveillance systems could provide reliable information. To this effect, the outputs in this analysis are based on a 22 year dataset from Butajira demographic surveillance site.

**Methods:**

The Butajira Rural Health Programme was launched in 1987 with an objective of developing and evaluating a system for a continuous registration of vital events. The surveillance system operates in an open cohort. An event history analysis was carried out to calculate the yearly neonatal mortality and its association with selected covariates. Poisson regression model was used to elicit neonatal mortality risk factors.

**Results:**

The trends of neonatal mortality equaled out at a higher level over the study period (P-value = 0.099). There was a high burden of early neonatal mortality (incidence rate ratio 4.8 [4.5, 5.2]) with the highest risk of death on the first day of life 18 [16.6, 19.4]. In multivariate analysis, males 1.6(1.4-1.9), neonates born from poor mothers who had no oxen 1.2(1.0-1.3) lived in thatched houses 2.9(2.4-3.5) and a distance to a health facility 1.5(1.1-2.0) conferred the highest risk of neonatal deaths.

**Conclusion:**

Despite an urgent need in reducing neonatal mortality which contributes to more than 40% to child mortality, no significant change was observed in Butajira. Death was significantly associated with sex of the child, socio-economic variables and physical access to hospital. Prevention strategies directed at reducing neonatal death should address policy and household and level factors, which significantly influence neonatal mortality in Butajira.

## Background

The neonatal period begins with birth and ends at 28 completed days after birth [1]. Neonatal deaths encompass early neonatal deaths which occur during the first seven days of life (0-6 days) and late neonatal deaths that occur after the seventh day but before the 28th day of life [2].

A large number of children may die soon after birth: many of them in the first four weeks of life (neonatal deaths), and most of those during the first week (early neonatal deaths). There are also cases where the babies are born dead; this is known as foetal death or stillbirth [3].

Almost all (99%) of neonatal deaths occur in low and middle-income countries, yet most epidemiological and other research focuses on only 1% of deaths which occur in rich, developed countries [4].

There are a high number of neonatal deaths reported from south-central Asian countries while Sub-Saharan Africa harbours the highest. In the period of 1990-2009, 31 million neonatal death happened in southeast Asian low and middle income countries, 21 million in African low and middle income countries and one million in higher income countries of Africa [5].

Globally, the proportion of child deaths that occurs in the neonatal period increased from 37% in 1990 to 41% in 2009 [6-8]. In order to achieve the world Millennium Development Goals of lowering the under-5 mortality rate of 1990 by two-thirds by 2015, it is critical to reduce neonatal mortality rates [9-13].

Ethiopia has a high child mortality rate, with large proportion of deaths occurring in the first month of life. Over the preceding 15 years infant and under-five mortality had underwent considerable improvement with a reduction in the rate of approximately 48 and 45 percent respectively. However, there is no marked improvement in neonatal mortality [13].

Globally, the main direct causes of neonatal death include preterm birth (28%), sepsis (26%), and asphyxia (23%). Neonatal tetanus accounts for a smaller proportion of deaths (7%), low birth weight is an important indirect cause of death but maternal complications in labour carry a high risk of neonatal death and poverty is also strongly associated with an increased risk [7,8].

Information on perinatal and late neonatal mortality is important to contribute to the effort towards reducing infant mortality [4]. In countries where there is no civil registration system, health and demographic surveillance data will serve to examine the trend and factors affecting neonatal mortality. In this study we assessed the trends of neonatal mortality and factors affecting it using the 22 years Butajira data set.

## Methods

### Study setting

We conducted this study in Butajira analysing the Butajira Rural Health Program (BRHP) 22 years data set in 2012.

Butajira Rural Health Programme is situated in the south central Ethiopia in Gurage Zone. The district is located 135 Km south of the capital Addis Ababa. The district has an estimated population of 175,682 [14]. The BRHP covers a sample within the district, following ten kebeles/villages initially sampled from the entire district using a probability proportional to size technique. At the time of the study, the BRHP covered about 70,000 individuals. Nine of the ten kebeles are rural while one is an urban kebele located in Butajira town. Guragigna is the predominant language [15-18].

### Nature of the BRHP

The BRHP was launched in 1986 with an objective of developing and evaluating a system for continuous registration of vital events, including birth, death, migration and internal movements, marriage and household characteristics. In this arrangement, the first census was conducted in April 1986. Data were collected initially monthly, later quarterly by visiting each household using village-based enumerator; each household is identified by unique number within its village, and each individual within their household [15].

### Data analysis

An Event History Analysis (EHA) was carried out using the Butajira HDSS 22 year data set. All 10 villages and all deaths of children under the age of 28 days that occurred between 1987 and 2008 were included in the analysis. Data analysis was conducted using STATA version 11 software. Mortality rates estimation was done using the number of deaths and person-days lived by the children for each year. These were computed from the date of entrance into the BRHP by birth and the date children exited by death. Unadjusted and adjusted neonatal mortality Incidence Rate Ratio (IRR) along with 95% CI and comparison of variables and risk factors of mortality were calculated using Poisson Regression Model. Reference categories were defined as those usually associated with the lowest neonatal mortality rates. All variables found to be significant in bivariate analysis were then included in a multivariate Poisson regression model and adjusted IRR with 95% confidence intervals were calculated. The approach used robust standard errors in order to manage the deviance and correlation created from longitudinal nature of the data. Overall time trends in mortality were analyzed and test of significance was checked by looking the 95% Confidence Interval (CI). Mortality curves were prepared by running mean smoother (with 4-years moving averages) in order to smooth random fluctuation in the material.

### Ethical issues

Ethical clearance was obtained from the research ethics committee of the School of Public Health at Addis Ababa University. Permission to use the database was obtained from BRHP Technical Management Committee. In addition, personal identifiers were removed from the data to maintain anonymity.

## Results

During the period 1987 through 2008, a total of 1055 deaths of children aged 0-27 days were identified and contributed 803,370 person-days. Of the 1055 neonatal deaths, 768 [73%] were early neonatal deaths (0-6 days) and 287 [27%] were late neonatal deaths (7-27 days). Of the total number of neonatal mortality 573 [54%] occurred in the first 24 hours of life.

An overall neonatal mortality incidence rate (NMIR) of 1.3 neonatal deaths per 1000 person-days (95% CI: 1.2, 1.4), early neonatal mortality incidence rate (ENMIR) of 4.8 early neonatal deaths per 1000 person-days (95% CI: 4.5, 5.2) and late neonatal mortality incidence rate (LNMIR) of 0.4 late neonatal deaths per 1000 person-days (95% CI: 0.4 0.5) was identified (Table 1). The incidence of dying in the first 24 hours after birth was 18 per 1000 person-days (95% CI: 17.0, 19.4).

**Table 1:** Distribution of the neonatal mortality incidence rate by selected differentials, Butajira district 1987-2008

**Variable**	**Age of death in days**		**Person-days,**		**Neonatal mortality rate NMIR**		**Overall NMIR**
	**0-6**	**7-27**	**Early neonatal**	**Late neonatal**	**Early NMIR**	**Late NMIR**	
**Overall**	768	287	158471	644899	4.8(4.5, 5.2)	0.4(0.4, 0.5)	1.3(1.2, 1.4)
**Sex**							
Male	467	166	80428	326581	5.8(5.3, 6.3)	0.5(0.4, 0.6)	1.5(1.4, 1.7)
Female	301	121	78043	318318	3.8(3.4, 4.3)	0.3(0.3, 0.4)	1.0(1.0, 1.2)
**Area**							
Lowland	360	142	62668	255120	5.74(5.2, 6.4)	0.6(0.5, 0.6)	1.6(1.4, 1.7)
High- land	324	119	68080	276470	4.76(4.2, 5.3)	0.4(0.3, 0.5)	1.3(1.2, 1.4)
Urban	84	26	27723	113309	3.0(2.4, 3.75)	0.2(0.1, 0.3)	0.8(0.6, 0.9)
**Religion**							
Muslim	386	133	101714	412817	3.8(3.4, 4.2)	0.3(0.3, 0.4)	1.0(0.9, 1.0)
Christian	86	41	29867	122065	2.9(2.3, 3.5)	0.3(0.2, 0.4)	0.8(0.7,1.0)
**House ownership**							
Own	682	268	141928	576599	4.8(4.4, 5.2)	0.5(0.4, 0.5)	1.3(1.2, 1.4)
Rented	39	7	11104	45806	3.5(2.5, 4.8)	0.1(0.1, 0.3)	0.8(0.6, 1.0)
Others	47	12	5439	22494	8.6(6.5, 11.5)	0.5(0.3, 0.9)	2.1(1.6, 2.7)
**Distance to hospital**							
<5 km	125	41	37420	152817	3.3(2.8, 4.0)	0.3(0.2, 0.4)	0.9(0.7,1.0)
5-9 km	302	118	59010	239741	5.1(4.6, 5.7)	0.5(0.4, 0.6)	1.4(1.3, 1.5)
> = 10 km	341	128	62041	252341	5.5(4.9, 6.1)	0.5(0.4, 0.6)	1.5(1.4, 1.6)
**Type of roof**							
Iron sheet	82	28	31790	129961	2.6(2.0, 3.2)	0.2(0.2, 0.3)	0.68(0.6,0.8)
Thatched	686	259	126681	514938	5.4(5.0, 5.8)	0.5(0.4,0.6)	1.5(1.4, 1.6)

A higher level of early neonatal mortality rate compared to late neonatal mortality rate was observed. There is a significant decline in mortality after neonates survive the first week of life in the observation period. The death of children on the first day comprises the larger proportion of early neonatal mortality. The excess male mortality rate was most prominent during the first week of life (0-6 days) and it was the approximate peak of the male disadvantage. After passing the first week of life mortality rate was at the same level for both sexes.

### Temporal trend of neonatal mortality in Butajira district (1987-2008)

**Figure 1:**
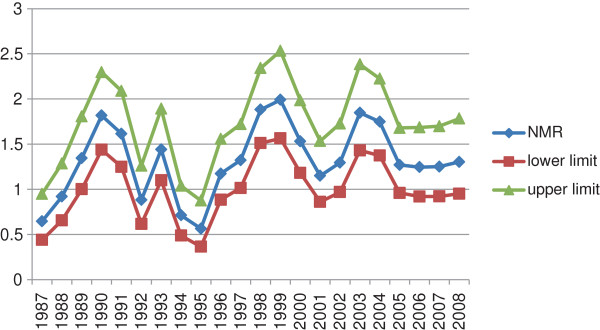
Observed rates by year in Butajira district 1987-2008.

Throughout this period, the year 1987-1991, the data has shown a significant increment for neonatal mortality trend (p-value = 0.02); while after the year 1992 the trend did not show any significant pattern. Nevertheless, the overall 22 years community based neonatal mortality curve showed a series of unstable rates - the confidence intervals around each rate are wide and overlaps each other and where the pattern over time is quite jagged (Figure 1).

Using a four year moving averages to gain stability and smoothness, no linear trend was observed for neonatal deaths over the years (P-value = 0.1) (Data not shown).

### Risk factors of neonatal mortality in the Butajira district (1987-2008)

A Poisson regression model, taking into account sex, residential area, source of water, house ownership, religion, type of roof and distance to hospital showed that male neonates had a significantly higher risk of death(adjusted incidence rate ratios [with 95% CI] 1.6 [1.4-1.9] than females. Mortality rates were significantly higher among Muslims compared to all Christian religion followers (adjusted incidence rate ratios [95% CI] 1.2 [1.0-1.5].

Neonates born to mothers who have no oxen have adjusted incidence rate ratio (95% CI) 1.2(1.0-1.3). Those who were born to mothers living in thatched housing were more likely to die (adjusted incidence rate ratio [95% CI] 2.9 [2.4-3.5]) than their counterparts. Neonates who were born to mothers living (5-9 km) away from Butajira hospital had an increased risk of dying (adjusted incidence rate ratio [95% CI] 1.5 [1.1-2.0]) than those who lived closer than 5 km (Table 2).

**Table 2:** Poisson regression model, depicting mortality rate ratios adjusted for different risk factors among neonates in Butajira district for the period (1987-2008)

**Variable**		**Person days**	**Unadjusted IRR (95% CI)**	**Adjusted IRR (95% CI)**
**Sex**				
Male	633	407009	1.6(1.4-1.8)	1.6(1.4-1.9)*
Female	422	396361	Ref.	Ref.
**Area**				
Low land	502	317788	2.8(2.3-3.5)	1.0(0.7 -1.8)
High land	443	344550	2.0(1.6-2.5)	0.8(0.5 -1.3)
Urban	110	141032	Ref.	Ref.
**Religion**				
Muslim	519	514531	1.3 (1.1-1.6)	1.2(1.1 -1.5)*
Christian	127	151932	Ref.	Ref.
**House ownership**				
Own	950	718527	2.0(1.5-2.7)	0.9(0.6-1.3)
Rented	46	56910	Ref.	Ref.
**Water source**				
Protected	296	277510	0.8 (0.7-0.9)	1.1(0.9-1.3)
Unprotected	759	525860	Ref.	Ref.
**Distance to hospital**				
<5 km	166	190237	Ref.	Ref.
5-9 km	420	298751	2.0(1.7-2.5)	1.5(1.1- 2.0)*
> = 10 km	469	314382	2.2(1.8-2.6)	1.3(0.9- 1.8)
**Oxen**				
No	787	534824	1.4 (1.2-1.6)	1.2(1.0- 1.3)
Yes	268	268546	Ref.	Ref.
**Roof**				
Thatched	945	641619	2.6(1.8-3.9)	2.9(2.4- 3.5)*
Iron sheet	110	161751	Ref.	Ref.

(Ref = reference category, RR = 1).

*Significant at significance level of 0.05.

*RR* Relative Risk.

## Discussion

Our data suggests that there was no significant decline observed in neonatal mortality trends in the study period. High mortality was associated with male sex, distance to hospital, born to mothers who have no oxen and neonates who were born to mothers living in thatched houses.

The mortality rate estimation using person days generated from the surveillance as denominator, indicated that the risk of dying in the early neonatal mortality was four times greater than in the late neonatal period. This is consistent with other studies where the majority of neonatal deaths happened at early neonatal period [4,16]. This could be because the majority of neonatal deaths are associated with events surrounding delivery, pregnancy, and neonatal care following birth. In rural Ethiopia including our study area most births occur at home [14].

Since the yearly trends of neonatal mortality showed a series of unstable rates, the confidence intervals around each rate was very wide and crossover each other, the pattern over time is quite jagged so it is very difficult to meaningfully interpret the yearly mortality rate. Therefore, to overcome the problem a four years moving average was used in order to get a smoother curve and enhance interpretation of the findings. However, the overall mortality trends did not show a change; this result is consistent with EDHS that states there was no considerable decline in neonatal mortality observed between 2005 and 2011 [13].

The sex of the neonates significantly influenced the rate of dying and is consistent with other studies. We found females had a lower risk of mortality than males during the first month of life [19-21]. This increased risk may also be due to the large proportions of neonatal deaths occurring in the first week, which is the time when gender differences in neonatal mortality are more pronounced [22]. The biological factors that have been implicated with these increased risks of neonatal deaths in male infants include respiratory syndrome related to late maturity [23] immunodeficiency [24] increasing the risks of infectious diseases in males, late maturity [22] resulting in a high prevalence of respiratory diseases in males, and congenital malformations of the urogenital system.

Recent studies revealed that distribution of neonatal mortality rates vary in rural-urban residence where neonatal mortality is high in rural areas [22-26]. In this study, it was clear that there was an evidence of change in mortality between urban and rural at bivariate analysis. However, the multivariate regression model suggests there were no persistent disparities between the residential area. This is consistent with EDHS 2011, which states urban areas have lower mortality than in rural areas 41 per 1000 live births and 43 per 1000 live births respectively [13]. Though the difference is quite small and is not statically significant it could be explained by progress made in ANC coverage in Ethiopian, because of the community health programmes newly implemented at grass root level.

In most countries, the mortality rates vary with socio-economic status [13,25,27]. In this study though we have incorporated some proxy indicators for socio-economic status such as type of roof, oxen and house ownership we could not compute a wealth index as we have very few variables with this regard. These socio-economic indicators suggested that neonates from families living in disadvantaged conditions were dying most. With this regard, neonates who were born to mothers who had no oxen, those who were living in thatched housing had a high neonatal mortality rate during the study period.

On the other hand though, house ownership is a positive indicator of wealth, this study indicated that neonates borne from parents who lived in their own houses were more likely to die than those who lived in rented houses. This is largely attributed to most people in the rural area own a substandard one room, earthen floor huts without ventilation and usually a common place for animals and humans together. While on the other hand those who live in rented houses may be people who are gainfully employed and usually living in urban areas. Religion emerged as the predictor of neonatal mortality. Those neonates born to Muslims significantly influenced the rate ratio of neonatal dying. This increased risk could be largely explained by large fixed religion effect on study area.

Relying on straight-line distance from home to the hospital as an indicator of geographical accessibility to hospital facilities utilizing GIS to estimate distance to hospital; potentially a more accurate assessment of geographical accessibility was assessed. There is increasing risk of neonatal death with long travel time to hospital in Butajira district. However, a distance further than 10 km had no significant effect on neonatal mortality. Larger numbers of episodes in this segment of population making the denominator to be big and the rate to become small could explain this. Therefore, there was increasing risk of neonatal death with long travel time to hospital. Our findings are consistent with those of previous studies that identified universal access to basic health services before, during and after childbirth as being protective against the occurrence of perinatal deaths [28]. Contrary to this study, other findings found that the longer the distance between maternal residences had no significant effects on neonatal mortality [29,30]. They suggest that factors other than geographic access may be crucial to understanding the risks associated with health care utilization. These could include quality of care, level of available care (primary versus secondary), cost and social barriers. Therefore, this suggests problems of service quality rather than geographic access and highlights the need to assess and improve the capacity of health facility.

A limitation of the study is that there is a potential to miss neonatal deaths, particularly early neonatal deaths, which would underestimate the overall neonatal mortality burden. Neonates that are born and die during the same day may not be reported, particularly if the mother migrated out of the DSS to have her maternity period with parents which is common in the study area especially for primigravidas. However, any missed deaths have always been incorporated into the database retrospectively when discovered as the data collectors are living in the villages. The other limitation is that some important determinants of neonatal mortality were not explored because of missing data and lack of information, this also could be seen as limitation of this study.

## Conclusion

Our findings demonstrated that no significant change was observed in neonatal mortality trends in the past 22 years. This suggests that the neonatal mortality which contributes more than a third of infant mortality was stable at higher level. This finding justifies that a lot of work needs to be done in order to decrease this high neonatal mortality rate by considering child health policy and household level factors, which significantly influence neonatal mortality in Butajira.

## Competing interests

The authors declare that they have no competing interests.

## Authors’ contributions

MG carried out the conception and designing the study, performed statistical analysis and wrote the manuscript. MM and WM participated in the data quality assurance, designing the study, analysis and write-up. All authors have approved the final form of the manuscript.

## Authors’ information

MG (MPH in Epidemiology), lecturer at the School of Public Health of Addis Ababa University, MM (MPH, PhD) Assistant Professor of Public Health in Addis Ababa University, WM (MA,PhD) working in Addis Ababa University School of Public Health.

## Pre-publication history

The pre-publication history for this paper can be accessed here:

http://www.biomedcentral.com/1471-2393/14/64/prepub

## References

[B1] Hill K Choi Y Neonatal mortality in the developing world Journal of Health Management 2006 14 18 429 452

[B2] WHO International Classification of Disease (ICD-10), Health Assembly 1990 10

[B3] WHO Neonatal and Perinatal Mortality: Country, Regional and Global Estimates 2006

[B4] Lawn JE Cousens S Zupan J For the lancet neonatal survival steering team neonatal survival Lancet 2005 365 891 900 10.1016/S0140-6736(05)71048-5 15752534

[B5] Oestergaard MZ Inoue M Yoshida S Mahanani WR Gore FM Neonatal mortality levels for 193 countries in 2009 with trends since 1990: a systematic analysis of progress, projections, and priorities PLoS Med 2011 8 8 e1001080 10.1371/journal.pmed.1001080 21918640 PMC3168874

[B6] Lawn JE Cousens S Zupan J For the lancet neonatal survival steering team neonatal survival Lancet 2005 365 896 900

[B7] Lawn JE Cousens S Zupan J 4 million neonataldeaths: When? Where? Why? Lancet 2005 365 891 900 10.1016/S0140-6736(05)71048-5 15752534

[B8] UNICEF Millennium development goals 2012 http://www.unicef.org/mdg/

[B9] World Health Organization The World Health Report 2005 Make Every Mother and Child Count 2005 Geneva: WHO

[B10] Child Mortality Coordination Group Tracking progress towards the millennium development goals: reaching consensus on child mortality levels and trends Bull WHO 2006 84 225e32 16583082 10.2471/blt.05.029744 PMC2627288

[B11] Black RE Morris SS Bryce J Where and why are 10 million children dying every year? Lancet 2003 361 2226e34 12842379 10.1016/S0140-6736(03)13779-8

[B12] UNICEF World declaration on the survival, protection and development of children http://www.unicef.org/wsc/declare.htm (Accessed Jan 2012)

[B13] Central Statistics Authority Ethiopia Demographic and Health Survey 2011

[B14] Woreda Health Office Meskan Woreda Information Desk 2011

[B15] Berhane Y Wall S Establishing an epidemiological field laboratory in ruaral areas-potential for public health research and intervention: the Butajira rural health programme 1987-99 Ethiop J Health Dev 1999 13 special issue

[B16] Byass P Fantahun M Emmelin A Molla M Berhane Y Spatio-Temporal Clustering of Mortality in Butajira, Ethiopia, from 1987–2008 2008 10.3402/gha.v3i0.5244 PMC2935921 20838630

[B17] Molla M Byass P Berhane Y LB Mortality decreases amoung young adults in southern centeral Ethiopia EthiopJHealth Dev 2008 22 3 218 225

[B18] Anders E Mesganaw F Yemane B Stig W Byass P Climate change and infectious diseases ;Vulnerability to episodes of extreme weather: Butajira, Ethiopia, 1998–1999 Global Health Action 2009 2 doi:10.3402/gha.v1i0.1829 10.3402/gha.v2i0.1829 PMC2799308 20052373

[B19] Christiana R Michael J Kingsley A Christine L Hall J Determinants of neonatal mortality in Indonesia BMC Public Health 2008 8 232 doi:10.1186/1471-2458-8-232 10.1186/1471-2458-8-232 PMC2478684 18613953

[B20] Khoury M Marks J McCarthy B Factors affecting the sex differential in neonatal mortality: the role of respiratory distress syndrome Am J Obstet Gynecol 1985 151 6 777 782 10.1016/0002-9378(85)90518-6 3976790

[B21] Green M The male predominance in the incidence of infectious diseases in children: a postulated explanation for disparities in the literature Int J Epidemiol 1992 21 2 381 386 10.1093/ije/21.2.381 1428496

[B22] Yanping W Lei M Li D Chunhua H Xiaohong L Mingrong L Rural urban differences in neonatal mortality rate in China, 1996e2006 J Epidemiologic Community Health 2010 64 935e6 10.1136/jech.2009.093138 20584731

[B23] Bin Y Li W Hong L Weimin F Yang H Youjie W Rural-urban differences of neonatal mortality in a poorly developed province of China BMC Public Health 2011 11 477 M.M 10.1186/1471-2458-11-477 21682907 PMC3144461

[B24] Hossain M Islam M Socio-economic variables affecting infants and children mortality in Bangladesh Internet J Health 2009 9 2 doi:10.5580/8f5

[B25] Carcillo JA Diegel JE Bartman BA Guyer FR Kramer SH Improved maternal and child healthcare access in a rural community J Health Care Poor Underserved 1995 1 23 40 7734633 10.1353/hpu.2010.0260

[B26] Pasquier C Morelle M Bagouet S Moret S Effects of residential distance to hospitals with neonatal surgery care on prenatal management and outcome of pregnancies with severe fetal malformations Ultrasound Obstet Gynecol 2007 29 271 275 10.1002/uog.3942 17318944

[B27] Sartorius B Kahn K Younatou P Collinson M Tollman S Young and vulnerable: Spatial temporal trends and risk factors for infant mortality in rural South Africa (Agincourt), 1992-2007 BMC Public Health 2010 10 645 10.1186/1471-2458-10-645 20977724 PMC3091567

[B28] Dummer B Parker L Hospital accessibility and infant death risk Arch Dis Child 2004 89 232 234 10.1136/adc.2003.030924 14977699 PMC1719848

[B29] Alonso V Fuster V Luna F Causes of neonatal mortality in Spain (1975–98): influence of sex, rural-urban residence and age at death J Biosoc Sci 2006 38 4 537 551 10.1017/S0021932005026957 16762089

[B30] NIPORT Bangladesh Maternal Health Services and Maternal Mortality Survey 2001 (English) 456

